# The magnitude of obesity and metabolic syndrome among diabetic chronic kidney disease population: A nationwide study

**DOI:** 10.1371/journal.pone.0196332

**Published:** 2018-05-09

**Authors:** Piyawan Kittiskulnam, Nintita Sripaiboonkij Thokanit, Pisut Katavetin, Paweena Susanthitaphong, Nattachai Srisawat, Kearkiat Praditpornsilpa, Kriang Tungsanga, Somchai Eiam-Ong

**Affiliations:** 1 Division of Internal Medicine-Nephrology, Department of Medicine, Faculty of Medicine, Chulalongkorn University and King Chulalongkorn Memorial Hospital, Thai Red Cross Society, Bangkok, Thailand; 2 Special Task force for Activating Research in Renal Nutrition (Renal Nutrition Research Group), Office of Research Affairs, Chulalongkorn University, Bangkok, Thailand; 3 Division of Nephrology, Department of Medicine, Faculty of Medicine, Chulalongkorn University, Bangkok, Thailand; 4 Ramathibodi Comprehensive Cancer Center, Ramathibodi Hospital, Mahidol University, Bangkok, Thailand; Istituto Di Ricerche Farmacologiche Mario Negri, ITALY

## Abstract

**Background:**

Although the prevalence of obesity and metabolic syndrome (MetS) among dialysis patients has been exceeding than general population, little is known regarding obesity and MetS in non-dialysis chronic kidney disease (CKD). We aimed to find the magnitude of obesity and MetS and their associations with impaired renal function among type 2 diabetes mellitus (T2DM) patients.

**Methods:**

A national survey of T2DM patients was collected in the Thai National Health Security Office database during 2014–5. The sampling frame was designated as distinct geographic regions throughout the country. A stratified two-stage cluster sampling was used to select the study population. Anthropometry and 12-hour fasting blood samples were obtained by trained personnel. BMI of ≥25 kg/m^2^ was classified as obesity. MetS was defined as having elevated waist circumference (>90 and >80 cm in men and women, respectively) plus any two of the followings: triglyceride ≥150 mg/dL, HDL-C <40 in men or <50 mg/dL in women, blood pressure ≥130/85 mmHg, and fasting blood sugar ≥100 mg/dL. CKD was defined as an impaired renal function (eGFR <60 mL/min/1.73m^2^ according to the CKD-EPI equation). Logistic regression analysis was performed to examine the relationship between obesity and MetS with the presence of CKD.

**Results:**

A total of 32,616 diabetic patients were finally recruited from 997 hospitals. The mean age was 61.5±10.9 years with 67.5% women. Of the participants, 35.4% were CKD patients. The prevalence of obesity was 46.5% in CKD and 54.1% in non-CKD patients with T2DM (*p*<0.001). In contrast, the prevalence of MetS in CKD patients was higher than their non-CKD counterparts (71.3 *vs* 68.8%, *p*<0.001). Moreover, there was an association between the prevalence of MetS with CKD stage from 3a to 5 (70.1, 72.3, 73.4, and 72.7%, respectively, *p trend* = 0.02). MetS, but not obesity, had a significant association with CKD in T2DM patients after adjusting for age, sex, and comorbidities [OR 1.14; 95% CI 1.06–1.22, *p*<0.001]. When stratified by each component of MetS, only high serum triglyceride and low HDL-C levels were increased in patients with CKD stage 4 and 5 compared with CKD stage 3 (*p*<0.001) and had a significant relationship with impaired renal function.

**Conclusion:**

There were relatively high prevalences of both obesity and MetS in T2DM patients. A higher prevalence of MetS, but lower prevalence of obesity, was observed among diabetic CKD group compared with their non-CKD counterparts. MetS, as a surrogate of insulin resistance, appeared to be more important than obesity in the development of impaired renal function in diabetic population.

## Introduction

Obesity is typically measured by the simple metric of body mass index (BMI) according to the World Health Organization (WHO) classification [1]. The prevalence of obesity is increasing worldwide and was observed in low- and middle-income countries as well [2]. An earlier study indicated that there was a substantially increased in obesity prevalence among general population of both sexes, all ages, and all races in the United States (U.S.) over the past decade [3]. Similarly, the third report of National Health and Nutrition Examination Survey (NHANES III) showed that metabolic syndrome (MetS) is highly prevalent among the U.S. residents and was subjected to be higher than that estimated over the past 10 years [4].

The concern of obesity as a major impact on the risk of developing incident chronic kidney disease (CKD) recently gained more attention among nephrologists. A large population survey documented that high BMI ranks as one of the strongest risk factors for new-onset decline in kidney function [5] with the higher risk ratio of almost twice compared with normal-weight individuals [6]. Also, a prospective cohort during the 9-year follow up period demonstrated that participants with MetS had a significantly higher risk of incident CKD after adjusting for the subsequent development of diabetes and hypertension and the strength of association seemed to increase as the numbers of components of MetS increased [7]. Moreover, data from a large epidemiological study showed that obesity was associated with doubled risk of CKD, which was further worsened by the presence of MetS [8].

A previous observational study indicated that the rate of obesity among incident dialysis patients doubled by a rate far exceeding the corresponding trends in the general population [9]. The United States Renal Data System (USRDS) database also revealed that the majority of patients at the time of dialysis initiation were obese [10] and the prevalence of MetS was as high as two-thirds [11]. However, scarce data are available regarding the magnitude of obesity and MetS in the setting of pre-dialysis CKD.

In recognition that diabetes ranked as the primary cause of the annual incident cases of end-stage renal disease (ESRD) [12, 13]. We conducted a nationwide study of type 2 diabetes mellitus (T2DM) patients and analyzed data that include measurements of height, weight, and waist circumference as well as other significant metabolic profiles. The goals of this study were to find the prevalence of obesity and MetS among diabetic CKD patients and to evaluate the associations of obesity, MetS, and its individual component as associated factors with the presence of impaired renal function.

## Materials and methods

### Study design and participants

This nationally representative cross-sectional study of patients with T2DM with or without preexisting hypertension was conducted to evaluate the standard of care among Ministry of Public Health hospitals participating in the National Health Security Office (NHSO)’s program throughout Thailand during 2014–2015. Eligible patients were over 20 years of ages and received regular follow-up visit during the past 12 months. Written informed consent was obtained from each participant before enrollment. All patients were recruited from the outpatient clinics. We used a two-stage cluster sampling to select study population. The sample size was derived from the proportion to size model. The sampling frame was designated as distinct geographic regions throughout the country. The sampling method was started at the first stage of 77 strata at the provincial level followed by the hospital level in each province. In each province, hospitals were stratified into 5 strata, according to size and number of health care providers at various levels. The largest size was regional center hospital providing more than 500 beds followed by provincial general hospital and large to small community hospitals. The tertiary care center and university hospitals were excluded from this study. All selected patients were invited by the study coordinators and nurses to minimize the selection bias. This study was approved by both the Ethical Review Committee for Research in Human Subjects, the Ministry of Public Health of Thailand and the Institutional Review Board of Chulalongkorn University (IRB No.354/60).

### Data collection, anthropometry, and laboratory assessment

Data were retrieved from patient’s medical records including demographic characteristics, co-morbidities using the 10^th^ revised version of International Classification of Disease (ICD-10), and laboratory results by the well-trained study coordinators. Data entry into the case record form was finally transferred to the central data management of the Consortium of the Thai Medical Schools (MedResNet) to adjudicate that the process of data collection was complied with the study protocol. The data management team was responsible for inquiries to study sites to verify the data. By a random selection process, site monitoring was performed approximately in 60 hospitals or 10% of study sites.

Study personnel measured height using a stadiometer and recorded weight to the nearest 0.1 kg. Waist circumference was determined using a measuring tape at umbilicus level while patient stood upright with arms at the sides during end of a normal expiration phase. We recorded the average of three consecutive systolic and diastolic blood pressure measurements. We calculated BMI using body weight divided by the square of height in meter. We collected a blood sample after 12-hour fasting period for serum creatinine, lipid profiles, fasting plasma glucose, and hemoglobin A1C level. A random, untimed urine albumin and creatinine ratio was also assessed.

### Outcome measures

The BMI values of ≥ 25, ≥ 23, and <18.5 kg/m^2^ were classified as obesity, overweight, and underweight among Asian population using the International Obesity Task Force criteria, respectively [14]. Central or abdominal obesity was defined as having elevated waist circumference (>90 and >80 cm in men and women, respectively) [15]. MetS was defined as the presence of central obesity plus any two of the following criteria of serum triglyceride level ≥150 mg/dL, high density lipoprotein cholesterol level (HDL-C) <40 mg/dL in men and <50 mg/dL women or receiving specific treatment for lipid abnormality, blood pressure ≥130/85 mmHg or previous diagnosis of hypertension, FPG ≥100 mg/dL or previous diagnosis with type 2 diabetes according to the International Diabetes Federation (IDF) [16].

Serum creatinine level and estimated glomerular filtration rate (eGFR) were determined as markers of kidney function. Derivation of eGFR was calculated by Chronic Kidney Disease Epidemiology Collaboration (CKD-EPI) 2009 equation [17]. CKD was defined as impaired renal function by eGFR of less than 60 mL/min/1.73m^2^ [18]. CKD stage 3a, 3b, 4, and 5 were defined as eGFR 45–59, 30–44, 15–29, and <15 mL/min/1.73m^2^ and not receiving renal replacement therapy, respectively. A spot albumin-to-creatinine ratio of 30 to 299 mg/g and >300 mg/g were considered as having microalbuminuria and macroalbuminuria, respectively [19].

### Statistical analysis

The prevalence of obesity, MetS, and its individual component were determined for the overall study sample. We described patient characteristics using mean ± standard deviation (SD) for normally distributed or median (interquartile range) for non-normally distributed variables. We compared patients’ characteristics using chi squared, unpaired t-tests, and Mann-Whitney U test as appropriate. Categorical data were presented in proportion or percentage. Differences between variables were compared using a one-way analysis of variance (ANOVA) and unpaired Student *t* tests for normally distributed variables. Kruskal—Wallis and Mann—Whitney *U* tests (Wilcoxon rank–sum test) were used to compare groups with non-normally distributed variables. We assessed the correlations between measures using Pearson’s correlation and the trend analysis using nonparametric test across the ordered groups was also performed. Selected variables were analyzed by both univariable and multivariable logistic regression (data presented as [odds ratio (OR); 95% confidence interval (CI)]). We examined a model that included age and sex (model 1). We then added coronary artery disease, cerebrovascular disease, left ventricular hypertrophy and peripheral arterial disease as comorbidities (model 2). We conducted all analyses in Stata 13 (StataCorp LP, College Station, TX), and *P* values less than 0.05 were considered statistically significant.

## Results

### Baseline demographic data of participants

A total of 32,616 patients with T2DM were finally recruited from 997 hospitals. Characteristics of the participants are shown in Table 1. The average age was 61.5 ± 10.9 years; 67.5% were women. The median duration of diabetes was 7.0 years (4–10 years). The mean FPG and hemoglobin A1C were 153.9 ± 55.8 mg/dL and 63 ± 1.0 mmol/mol, respectively. Twenty-two percent of participants received subcutaneous insulin injection. The most commonly prescribed anti-diabetic medication was biguanides (72.2%). The prevalence of obesity by BMI in the entire population was 51.5% (68.2% in women and 31.8% in men, *p* = 0.01). The prevalence of MetS (69.8%) was higher than that of obesity among T2DM patients and was also significantly different between both sexes (79.4% in women *vs* 48.8% in men, respectively, *p*<0.001).

**Table 1:** Patient characteristics.

Parameters	Total (n = 32,216)	Non-CKD group (eGFR≥60 ml/min, n = 21,544)	CKD group (eGFR<60 ml/min, n = 10,672)	*P* value†
Age, years	61.5±10.9	58.5±10.4	67.2±9.8	<0.001
Female, %	67.5	66.7	68.6	0.001
Duration of diabetes, years	7.0 (4–10)	6.0 (4–12)	8.0 (5–12)	<0.001
Hypertension, %	78.2	74.4	86.9	<0.001
Microalbuminuria, %	11.8	11.1	14.4	<0.001
Macroalbuminuria, %	5.3	4.1	8.3	<0.001
Receiving ACEI, %	39.1	41.4	36.1	<0.001
Receiving ARB, %	16.1	15.4	17.7	<0.001
Systolic blood pressure, mmHg	131.7±16.1	130.7±15.4	133.3±17.1	<0.001
Diastolic blood pressure, mmHg	74.6±10.3	75.3±10.0	73.1±10.6	<0.001
Body weight, kg	63.9±13.0	64.9±13.2	61.9±12.5	<0.001
Height, cm	157.6±7.9	157.9±7.9	156.9±7.8	<0.001
BMI, kg/m^2^	25.7±4.6	25.9±4.7	25.1±4.5	<0.001
WC, cm				
- Men	89.8±11.6	89.8±11.9	89.9±11.2	0.69
- Women	88.7±11.7	87.9±11.7	87.4±11.6	0.01
Blood urea nitrogen, mg/dL	17.3±10.8	13.7±6.8	23.3±13.2	<0.001
Serum creatinine, mg/dL	1.1±0.9	0.8±0.2	1.7±1.4	<0.001
eGFR, mL/min	70.9±26.9	86.9±16.7	41.6±13.5	<0.001
FPG, mg/dL	153.9±55.8	155.5±53.6	151.1±59.7	<0.001
HbA1C, mmol/mol	63.0±1.0	64.0±1.0	63.0±2.0	<0.001
Total cholesterol, mg/dL	186.9±47.9	186.4±47.9	187.8±47.8	0.02
LDL cholesterol, mg/dL	108.5±37.8	108.6±37.4	108.3±38.3	0.54
HDL cholesterol, mg/dL	47.6±15.4	48.4±15.1	46.1±15.7	<0.001
Triglyceride, mg/dL	169.5±107.0	163.5±103.2	180.2±110.8	<0.001
Hemoglobin, g/dL	12.1±2.7	12.6±2.1	11.4±3.2	<0.001
Serum uric acid, mg/dL	5.9±2.2	5.4±1.7	6.9±2.5	<0.001
Stratification of nutritional status*				
- underweight (BMI<18.5), %	3.6	3.2	4.3	<0.001
- normal weight (BMI 18.5–22.9), %	25.5	23.4	29.0	<0.001
- overweight (BMI 23–24.9), %	19.4	19.2	20.1	<0.001
- obese (BMI ≥ 25), %	51.5	54.1	46.5	<0.001
- BMI ≥ 27, %	33.8	36.1	29.5	<0.001
- BMI ≥ 30, %	15.6	17.1	12.8	<0.001
Prevalence of metabolic syndrome**, %	69.8	68.8	71.3	<0.001
- Abdominal obesity, %	69.8	68.8	71.3	<0.001
- Elevated blood pressure, %	88.9	86.7	93.9	<0.001
- High serum triglyceride level, %	46.2	43.2	51.8	<0.001
- Low HDL-cholesterol level, %	44.4	40.9	48.4	<0.001

BMI, body mass index (kg/m^2^); BSA, body surface area; BW, body weight; eGFR, estimated glomerular filtration rate by CKD EPI 2009 equation; HDL, High Density Lipoprotein; Kcal, Kilocalorie; FPG, fasting plasma glucose; LDL, Low Density Lipoprotein; HDL, high density lipoprotein; LDL, low density lipoprotein; WC, waist circumference. Data are presented as mean (SD) and median (25^th^ to 75^th^).

^†^*p*<0.05 consider significantly different between non-CKD and CKD groups.

*According to International Obesity Task Force criteria for Asian population

**According to the International Diabetes Federation

### Comparison of obesity and MetS between CKD and non-CKD populations

Thirty-five percent of the participants were CKD patients (n = 10,672). The average eGFR of diabetic CKD and non-CKD group were 41.6 ± 13.5 *vs* 86.9 ± 16.7 mL/min/1.73m^2^, respectively. The proportion of T2DM patients with eGFR of 45–59, 30–44, 15–29, and less than 15 mL/min/1.73m^2^ were 17.3%, 11.6%, 4.6%, and 1.9%, respectively. The diabetic CKD patients were older, had longer duration of disease, as well as higher proportion of having pre-existing hypertension and albuminuria. The mean BMI and waist circumference in diabetic CKD patients were lower than in non-CKD group (25.1 ± 4.5 *vs* 25.9 ± 4.7 kg/m^2^, *p*<0.001 and 88.2 ± 11.6 vs 88.5 ± 11.8 cm., *p* = 0.03, respectively). The degree of positive correlation between BMI and waist circumference did not differ between CKD (*r* = 0.65, *p*<0.001) and non-CKD groups (*r* = 0.66, *p*<0.001).

**Fig 1:**
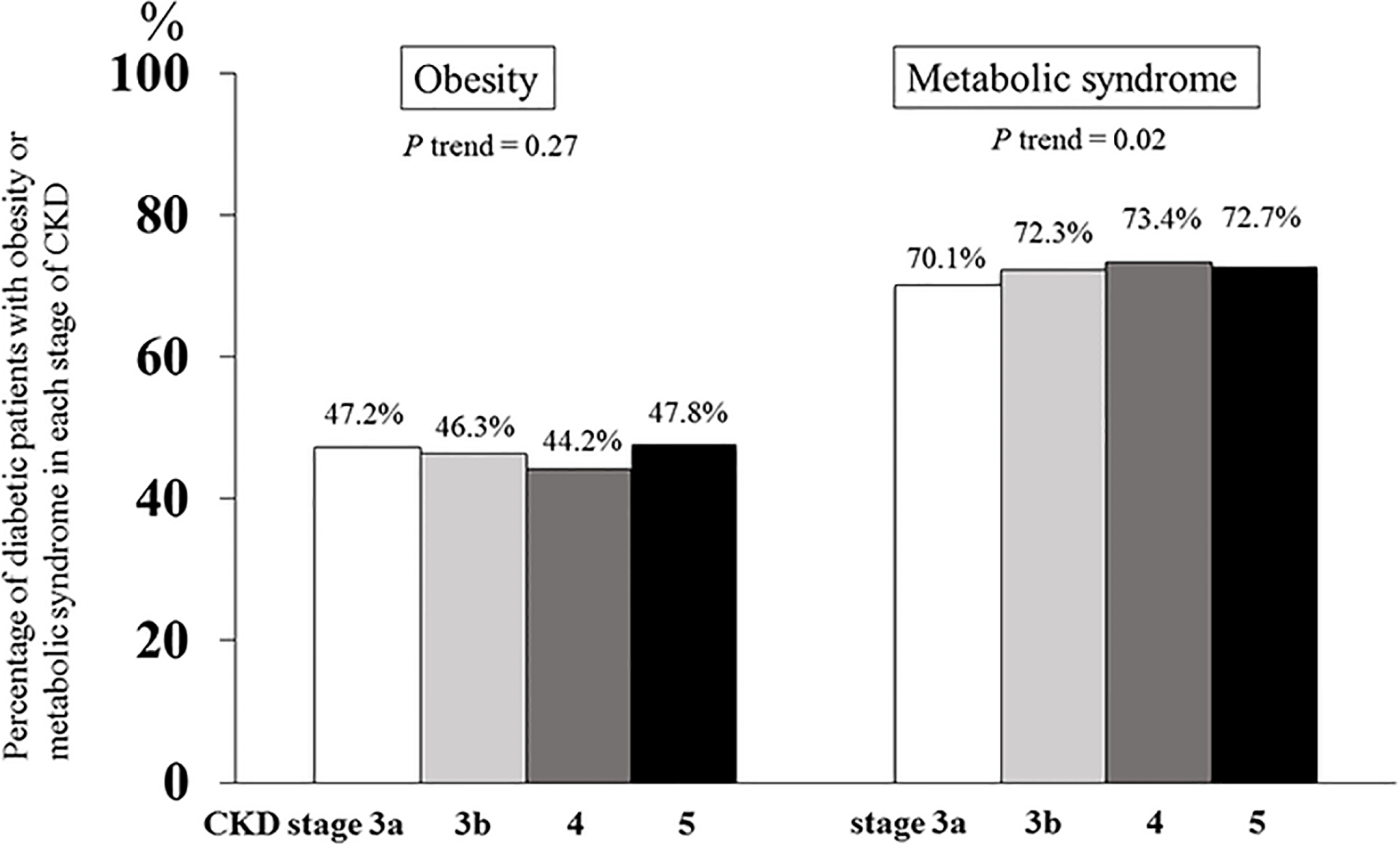
Percentage of type 2 diabetic patients with obesity and metabolic syndrome in each stage of chronic kidney disease (CKD). *P* trend <0.05 indicates statistical significance among CKD stage 3a, 3b, 4, and 5. White, light grey, dark grey, and black bars represent CKD stage 3a, 3b, 4, and 5, respectively.

The prevalence of obesity (BMI ≥25 kg/m^2^) was lower in CKD than non-CKD patients with T2DM (46.5% *vs* 54.1%, *p*<0.001) and there was no significant association between the prevalence of obesity and stages of CKD. In contrast, the prevalence of MetS in CKD was higher than non-CKD patients (71.3% *vs* 68.8%, *p*<0.001) (Table 1). Moreover, the percentage of patients with MetS increased from stage 3a to 4 among diabetic CKD patients (70.1%, 72.3%, 73.4%, and 72.7% of T2DM patients in CKD stage 3a, 3b, 4, and 5, respectively, *p* trend = 0.02) (Fig 1). The difference in prevalence of MetS and obesity was larger in CKD than non-CKD group (24.8% *vs* 14.7%). When stratified by age and sex in diabetic CKD group, the presence of MetS was significantly higher among female compared with male (79.8% *vs* 51.6%, *p*<0.001) as well as younger age than elderly diabetic patients (72.8% *vs* 70.4%, *p* = 0.03).

### Factors associated with the presence of impaired renal function among diabetic patients

When compared to patients with normal BMI, both overweight and obese patients had significantly lower risk for the presence of reduced renal function in the unadjusted analysis (OR 0.73; 95% CI 0.69–0.77, *p*<0.001 and OR 0.74; 95% CI 0.70–0.77, *p*<0.001, respectively). However, these associations had no longer statistical significance after adjustment for confounders. In addition, there was no significant association of BMI as continuous values with the presence of CKD after adjusting for age, sex, and comorbidities (OR 0.99; 95% CI 0.99–1.004, *p* = 0.64 for 1 kg/m^2^ increase in BMI).

According to the subtypes of classification of obesity and MetS, the majority of participants in this study were simultaneously having both obesity and MetS (metabolically unhealthy obese group, 47.2%). There were 22.6% of patients diagnosed with MetS but did not have concurrent obesity (metabolically unhealthy non-obese) and 5.9% with metabolically healthy obese (patients with obesity but not MetS). When we further analyzed data, metabolically unhealthy non-obese (OR 1.18; 95% CI 1.08–1.29, *p*<0.001), but not metabolically healthy obese group (OR 0.99; 95% CI 0.86–1.16, *p* = 0.98), was significantly associated with eGFR <60 mL/min/1.73m^2^ in the multivariable models compared to metabolically heathy non-obese group as a reference (Table 2).

**Table 2:** Unadjusted and adjusted odd ratio (OR) and 95% confidence interval (CI) for the relationship between different subclasses of obesity and metabolic syndrome with the presence of CKD.

Methods	Unadjusted		Model 1		Model 2	
	OR (95% CI)	P	OR (95% CI)	P	OR (95% CI)	P
The classification of obesity and metabolic syndrome according to different subclasses						
-Metabolically healthy non-obese group	reference		reference		reference	
-Metabolically healthy obese group	0.75 (0.66–0.86)	<0.001	1.00 (0.86–1.16)	0.99	0.99 (0.86–1.16)	0.98
-Metabolically unhealthy non-obese group	1.39 (1.28–1.51)	<0.001	1.19 (1.09–1.30)	<0.001	1.18 (1.08–1.29)	<0.001
-Metabolically unhealthy obese group	0.94 (0.87–1.01)	0.08	1.13 (1.05–1.23)	0.02	1.12 (1.04–1.22)	0.004

Model 1 adjusted for age and sex. Model 2 further adjusted for comorbidities (coronary artery disease, cerebrovascular disease, left ventricular hypertrophy, and peripheral arterial disease). Metabolically healthy non-obese group defined as individuals without obesity and metabolic syndrome. Metabolically healthy obese group defined as individuals with obesity but not concurrent metabolic syndrome. Metabolically unhealthy non-obese group defined as individuals with metabolic syndrome but not concurrent obesity. Metabolically unhealthy obese group defined as individuals with both metabolic syndrome and obesity.

MetS and its components contributed to the risk for the presence of impaired renal function. Diabetic patients with MetS had a significant higher risk associated with the presence of CKD (OR 1.13; 95% CI 1.06–1.20, *p*<0.001) in unadjusted analysis and this association persisted after adjustment for confounding factors (OR 1.14; 95% CI 1.06–1.22, *p*<0.001). Among individual criterion of MetS, the presence of abdominal obesity did not show a significant relationship with CKD in the adjusted models (Table 3). In contrast, having abnormal lipid profiles and elevated blood pressure demonstrated a significant association with eGFR of less than 60 mL/min/1.73m^2^, with the highest odds of impaired renal function for elevated serum triglyceride level (OR 1.65; 95% CI 1.56–1.74, *p*<0.001) followed by high blood pressure level (OR 1.55; 95% CI 1.40–1.70, *p*<0.001), and low HDL-C level (OR 1.35; 95% CI 1.28–1.42, *p*<0.001) in the final adjusted model of multivariable regression analysis.

**Table 3:** Unadjusted and adjusted odd ratio (OR) and 95% confidence interval (CI) for the association of metabolic syndrome and its individual component with the presence of CKD.

Methods	Unadjusted		Model 1		Model 2	
	OR (95% CI)	P	OR (95% CI)	P	OR (95% CI)	P
The presence of MetS and its individual component†						
-Metabolic syndrome*	1.13 (1.06–1.20)	<0.001	1.15 (1.07–1.24)	<0.001	1.14 (1.06–1.22)	<0.001
-WC >90 cm in men and >80 cm in women	0.97 (0.92–1.03)	0.29	0.99 (0.93–1.05)	0.68	0.97 (0.92–1.04)	0.52
-blood pressure ≥ 130/85 mmHg	2.39 (2.19–2.62)	<0.001	1.57 (1.43–1.73)	<0.001	1.55 (1.40–1.70)	<0.001
-serum triglyceride level ≥ 150 mg/dL	1.41 (1.34–1.48)	<0.001	1.65 (1.57–1.75)	<0.01	1.65 (1.56–1.74)	<0.001
-serum HDL-C <40 mg/dL in men and <50 mg/dL in women	1.36 (1.29–1.42)	<0.001	1.34 (1.29–1.43)	<0.01	1.35 (1.28–1.42)	<0.001

BMI, body mass index; HDL-C, high density lipoprotein cholesterol; WC, waist circumference. Model 1 adjusted for age and sex. Model 2 further adjusted for comorbidities (coronary artery disease, cerebrovascular disease, left ventricular hypertrophy, and peripheral arterial disease).

*metabolic syndrome was defined as having elevated waist circumference (>90 and >80 cm in men and women, respectively) plus any 2 of the followings: triglyceride>150 mg/dL, HDL<40 in men or <50 mg/dL in women or receiving lipid-lowering drugs, BP≥130/85 mmHg or receiving anti-hypertensive drugs, and blood sugar ≥100 mg/dL or being diagnosed with diabetes mellitus.

^†^Since all patients were type2 diabetes, we did not analyze the ORs for elevated plasma glucose criterion of metabolic syndrome.

### Characteristics of each component of MetS between early and late stage of CKD

**Fig 2:**
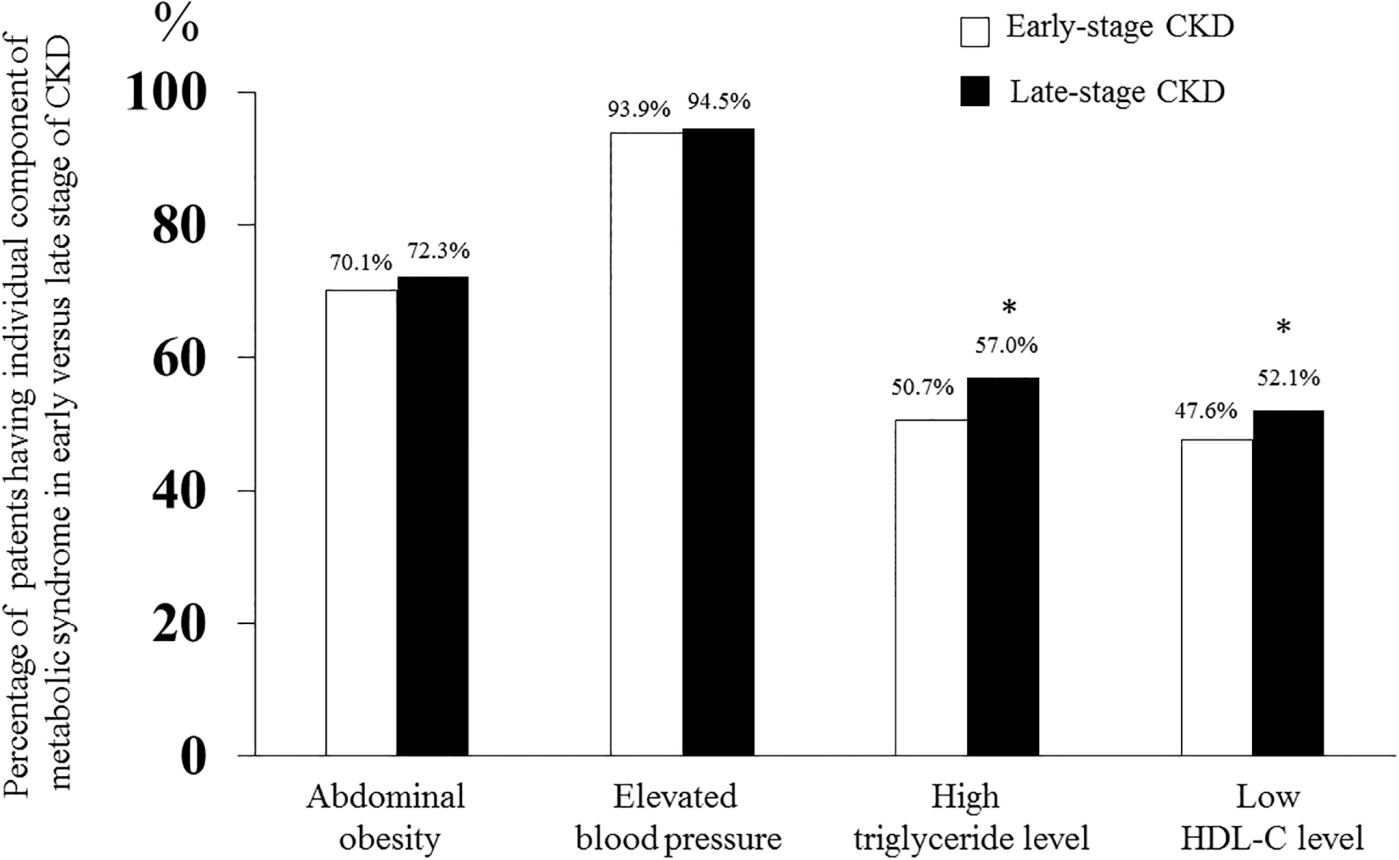
Percentage of each component of metabolic syndrome (abdominal obesity, elevated blood pressure, high serum triglyceride level and low high-density lipoprotein cholesterol) between early (30–59 mL/min/1.73m^2^) compared with late (less than 30 mL/min/1.73m^2^) diabetic CKD subgroup. * *P* <0.05 consider significant difference between late and early stage of CKD.

For individual component of MetS in diabetic CKD group, elevated serum triglyceride and low HDL-C levels significantly increased with the higher stage of CKD compared with CKD stage 3 (*p* <0.001) (Fig 2), whereas the proportion of patients with abdominal obesity as well as elevated blood pressure in late stage of CKD seemed to be higher than the early-phase CKD but it did not reach statistical significance (70.1% *vs* 72.3%, *p* = 0.10 and 93.9% *vs* 94.5%, *p* = 0.32, respectively).

## Discussion

The results in the present study demonstrated that both obesity and MetS were prevalent in patients with T2DM. In diabetic CKD patients, a higher prevalence of MetS, but lower prevalence of obesity, was observed compared with their non-CKD counterparts. MetS, as a surrogate of insulin resistance, rather than obesity was significantly associated with the presence of impaired renal function among diabetic population.

In this large scale population-based study in Thailand, we demonstrated that diabetic individuals with obesity were approximately half of the entire participants (51.5%). This finding supports a bidirectional relationship that pre-existing obesity is a prominent behavioral-related risk for developing T2DM [20] whereas abnormality in insulin secretion might concomitantly potentiate the risk for subsequent obesity, known as the term ‘diabesity’ [21]. However, we believe that the actual prevalence of obesity might be underestimated due to the poor diagnostic performance of BMI to classify patients with subclinical obesity (normal BMI but excess body fat), particularly among CKD population [22]. In contrast to typical obesity, the central or abdominal fat which exerts more metabolically active part of adipose tissue is independently associated with MetS [23] and there was an observation that a certain subset of individuals, who despite having normal weight, was likely to have increased risk of abdominal fat accumulation, particularly among Asian in origin [24]. Therefore, we used the definition of MetS by the IDF for the main outcome analysis to emphasize the presence of abdominal obesity as an obligatory component for diagnosis. In the present study, there was a much higher prevalence of MetS (69.8%) compared to obesity prevalence with one-fifth of patients with metabolically unhealthy non-obese. The effect of progressive CKD, however, might potentiate the development of some components of MetS such as elevated blood pressure or altered plasma lipid metabolism that may result in an overestimation of MetS prevalence while the influence of muscle wasting-associated with CKD might underestimate the prevalence of obesity by BMI among CKD population as well. Given that BMI does not differentiate sex-specific variations in segmental body fat distribution [25], other alternative methods of obesity assessment such as waist circumference, waist-hip ratio, or conicity index is needed in order to identify T2DM patients with normal or relatively low BMI at risk for having occult metabolic abnormalities.

The prevalence of MetS was higher in diabetic CKD than non-CKD group, especially among women. The variations in the distribution in body shape, that make women tended to accumulate more fat below the waist as opposed to men, would impact the large differences in MetS prevalence among both sexes. In addition, we found an increased association in the prevalence of MetS from stage 3a to 4 diabetic CKD whereas this relationship was not observed with respect to the prevalence of obesity. An earlier study proposed hyperinsulinemia, that finally results in insulin resistance, as a fundamental pathway of MetS-induced kidney disease [26]. This hypothesis was demonstrated in an experimental study in participants with different categories of GFR by Kobayashi *et al*. [27] that insulin sensitivity, determined by glucose disposal rate, diminished at the earlier stage of CKD compared to healthy subjects and declined progressively to the degree of renal function impairment. Several plausible mechanisms linking insulin resistance to kidney dysfunction include hyperinsulinemia, adipocytokine dysregulation, and low-grade chronic inflammation, all of which eventually result in podocyte injury-induced albuminuria and profibrotic states [28].

A different population and various ethnicities can produce varying results when evaluating risk for CKD. Currently, there is still lacking evidence for a population with T2DM, which has heterogeneous and less predictable course of disease. Our data indicated that higher BMI seemed not to be related with CKD, whereas having MetS was significantly associated with the presence of impaired renal function after adjustment for confounders. This observation suggests that the relationship between obesity and renal dysfunction may be mainly mediated by metabolic abnormalities such as hypertension and plasma lipid abnormalities. We considered that the lack of association between BMI and the presence of CKD in our study could be due in part to the adjustment for cardiovascular-related comorbidities. Although the U.S. and European general population with obesity are at increased risk of kidney diseases [6], some studies reported that the association of obesity with CKD was not significant after adjusting for various cardiometabolic risk factors [29, 30]. A recent meta-analysis also revealed that the risks of CKD increased parallel with the level of BMI only among obese individuals with normal metabolic features [31]. In agreement with our results, Thomas and colleagues showed that having MetS in middle-aged healthy people from Asia and Western countries were correlated with an increased risk for the development of eGFR of less than 60 mL/min/1.73m^2^ [32]. A 5-year prospective analysis in Hong Kong suggested that the presence of MetS independently predicted the new onset of CKD in T2DM patients as well [33]. In fact, there are some indications that waist circumference and the cluster of abnormalities related to MetS, but not BMI, were strongly associated with the risk of arteriosclerosis that finally leads to altered local intrarenal hemodynamics [34, 35] and albuminuria [36] in diabetic patients. Furthermore, the systemic effects of excess visceral abdominal fat content, elevated blood pressure, and dyslipidemia might worsen kidney function as well [37]. Therefore, MetS as a surrogate for insulin resistance would be superior to BMI in determining type 2 diabetic patients with impaired renal function.

It was well-established that the higher risk for developing of CKD was proportional to the cumulative numbers of MetS components [34, 38]. However, earlier studies on the individual trait of MetS with kidney outcomes were not widely described. In our analysis, we did not find a significant relationship of abdominal obesity with reduced GFR and its prevalence between early *vs* late stage of CKD. Given that waist circumference is a commonly used method to assess abdominal obesity, it does not correctly differentiate between visceral and subcutaneous abdominal adipose tissue. A previous study by Franca and colleague [39]. found that the amount of visceral adipose tissue was more strongly associated with reduction of cystatin C-based GFR among patients with early stages of CKD than total abdominal obesity measured by waist circumference. Our result has been affirmed with the previous evidence [40] that high blood pressure is a strong predictor of reduced GFR. Furthermore, the findings by De Cosmo *et al*. [41] and Viazzi *et al*. [42] were congruent with our study that the reduction of eGFR below 60 mL/min/1.73m^2^ in T2DM patients was more likely to be associated with plasma lipid abnormalities. This data favored the hypothesis that atherogenic dyslipidemia, characterized by elevated triglyceride and to a lesser extent low HDL-C level, might initiate endothelial dysfunction and eventually result in microvascular complication of diabetes [43]. Taken together, a simple measure of central obesity such as waist circumference, as surrogate of visceral adiposity, combined with evaluation of other metabolic profiles are better predictors of impaired renal function than the traditional BMI metric. However, in-depth analysis of body composition to discriminate the visceral and subcutaneous abdominal adipose tissue is warranted in order to determine the pathogenic role of excess abdominal fat with renal outcomes.

Some strengths as well as limitations of our study should be taken into account. The present study was a large, homogenous geographical distribution of the participating centers, and well-designed study representing a nationwide study of T2DM patients in Thailand. In addition, we used a standard protocol for the measurement of waist circumference, as an indicator for central obesity, as well as laboratory assessment including serum creatinine level. However, we considered that our study was a cross-sectional design, therefore the association do not imply the causality. Prospective cohorts are required to ascertain the impact of MetS on progressive renal function decline. While our study used the IDF criteria for MetS, some utilized other criteria. Therefore, we included the analyses using the Joint Statement criteria for MetS [44] in S1–S3 Tables and found that the main results did not change. Finally, our data may not be applicable to other ethnicity of population with T2DM, which partly limits generalizability.

In conclusion, the relatively high prevalences of both obesity and MetS were observed among diabetic population. A higher prevalence of MetS, but lower prevalence of obesity, was observed among diabetic CKD group compared with their non-CKD counterparts. MetS, rather than obesity, was associated with the presence of impaired renal function in diabetic population.

## Supporting information

S1 Table Patient characteristics and the prevalence of MetS using the Joint Statement criteria. (DOCX)

S2 Table Unadjusted and adjusted odd ratio (OR) and 95% confidence interval (CI) for the relationship between different subclasses of obesity and metabolic syndrome using the Joint Statement criteria with the presence of CKD. (DOCX)

S3 Table Unadjusted and adjusted odd ratio (OR) and 95% confidence interval (CI) for the association of metabolic syndrome using the Joint Statement criteria and its individual component with the presence of CKD. (DOCX)
