# Supplementary material for: The magnitude of obesity and metabolic syndrome among diabetic chronic kidney disease population: A nationwide study
Source: PLoS One. 2018 May 9;13(5):e0196332. doi: 10.1371/journal.pone.0196332 (PMC5942778; doi:10.1371/journal.pone.0196332)
Supplement: S1 Table — (DOCX) [file pone.0196332.s001.docx]

**S1 Table.** Patient characteristics and the prevalence of MetS using the Joint Statement criteria.

| **Parameters** | **Total**  **(n=32,216)** | **Non-CKD group (eGFR≥60 ml/min,**  **n=21,544)** | **CKD group**  **(eGFR<60 ml/min,**  **n=10,672)** | ***P***  **value**† |
| --- | --- | --- | --- | --- |
| Age, years | 61.5±10.9 | 58.5±10.4 | 67.2±9.8 | <0.001 |
| Female, % | 67.5 | 66.7 | 68.6 | 0.001 |
| Duration of diabetes, years | 7.0 (4-10) | 6.0 (4-12) | 8.0 (5-12) | <0.001 |
| Hypertension, % | 78.2 | 74.4 | 86.9 | <0.001 |
| Microalbuminuria, % | 11.8 | 11.1 | 14.4 | <0.001 |
| Macroalbuminuria, % | 5.3 | 4.1 | 8.3 | <0.001 |
| Receiving ACEI, % | 39.1 | 41.4 | 36.1 | <0.001 |
| Receiving ARB, % | 16.1 | 15.4 | 17.7 | <0.001 |
| Systolic blood pressure, mmHg | 131.7±16.1 | 130.7±15.4 | 133.3±17.1 | <0.001 |
| Diastolic blood pressure, mmHg | 74.6±10.3 | 75.3±10.0 | 73.1±10.6 | <0.001 |
| Body weight, kg | 63.9±13.0 | 64.9±13.2 | 61.9±12.5 | <0.001 |
| Height, cm | 157.6±7.9 | 157.9±7.9 | 156.9±7.8 | <0.001 |
| BMI, kg/m^2^ | 25.7±4.6 | 25.9±4.7 | 25.1±4.5 | <0.001 |
| WC, cm  - men  - women | 89.8±11.6  88.7±11.7 | 89.8±11.9  87.9±11.7 | 89.9±11.2  87.4±11.6 | 0.69  0.01 |
| Blood urea nitrogen, mg/dL | 17.3±10.8 | 13.7±6.8 | 23.3±13.2 | <0.001 |
| Serum creatinine, mg/dL | 1.1±0.9 | 0.8±0.2 | 1.7±1.4 | <0.001 |
| eGFR, mL/min | 70.9±26.9 | 86.9±16.7 | 41.6±13.5 | <0.001 |
| FPG, mg/dL | 153.9±55.8 | 155.5±53.6 | 151.1±59.7 | <0.001 |
| HbA1C, mmol/mol | 63.0±1.0 | 64.0±1.0 | 63.0±2.0 | <0.001 |
| Total cholesterol, mg/dL | 186.9±47.9 | 186.4±47.9 | 187.8±47.8 | 0.02 |
| LDL cholesterol, , mg/dL | 108.5±37.8 | 108.6±37.4 | 108.3±38.3 | 0.54 |
| HDL cholesterol, mg/dL | 47.6±15.4 | 48.4±15.1 | 46.1±15.7 | <0.001 |
| Triglyceride, mg/dL | 169.5±107.0 | 163.5±103.2 | 180.2±110.8 | <0.001 |
| Hemoglobin, g/dL | 12.1±2.7 | 12.6±2.1 | 11.4±3.2 | <0.001 |
| Serum uric acid, mg/dL | 5.9±2.2 | 5.4±1.7 | 6.9±2.5 | <0.001 |
| Stratification of nutritional status*  - underweight (BMI<18.5), %  - normal weight (BMI 18.5-22.9), %  - overweight (BMI 23-24.9), %  - obese (BMI ≥ 25), %  - BMI ≥ 27, %  - BMI ≥ 30, % | 3.6  25.5  19.4  51.5  33.8  15.6 | 3.2  23.4  19.2  54.1  36.1  17.1 | 4.3  29.0  20.1  46.5  29.5  12.8 | <0.001  <0.001  <0.001  <0.001  <0.001  <0.001 |
| Prevalence of metabolic syndrome**, % | 54.4 | 52.1 | 58.7 | <0.001 |
| - Abdominal obesity, % | 69.8 | 68.8 | 71.3 | <0.001 |
| - Elevated blood pressure, % | 88.9 | 86.7 | 93.9 | <0.001 |
| - High serum triglyceride level, % | 46.2 | 43.2 | 51.8 | <0.001 |
| - Low HDL-cholesterol level, % | 44.4 | 40.9 | 48.4 | <0.001 |

BMI, body mass index (kg/m^2^); BSA, body surface area; BW, body weight; eGFR, estimated glomerular filtration rate by CKD EPI 2009 equation; HDL, High Density Lipoprotein; Kcal, Kilocalorie; FPG, fasting plasma glucose; LDL, Low Density Lipoprotein; HDL, high density lipoprotein; LDL, low density lipoprotein; WC, waist circumference

Data are presented as mean (SD) and median (25^th^ to 75^th^). †*p*<0.05 consider significantly different between non-CKD and CKD groups. *According to International Obesity Task Force criteria for Asian population **According to the Joint Statement criteria of MetS.
