# Supplementary material for: The magnitude of obesity and metabolic syndrome among diabetic chronic kidney disease population: A nationwide study
Source: PLoS One. 2018 May 9;13(5):e0196332. doi: 10.1371/journal.pone.0196332 (PMC5942778; doi:10.1371/journal.pone.0196332)
Supplement: S2 Table — (DOCX) [file pone.0196332.s002.docx]

**S2 Table.** Unadjusted and adjusted odd ratio (OR) and 95% confidence interval (CI) for the relationship between different subclasses of obesity and metabolic syndrome using the Joint Statement criteria with the presence of CKD.

| **Methods** |  | **Unadjusted** | |  | **Model 1** | |  | | **Model 2** | | | |  | |
| --- | --- | --- | --- | --- | --- | --- | --- | --- | --- | --- | --- | --- | --- | --- |
|  |  | OR  (95% CI) | P |  | OR  (95% CI) | P |  | | OR  (95% CI) | | P | |  | |
| **The classification of obesity and metabolic syndrome according to different subclasses** | | | | | | | | | | | | | | |
| -Metabolically healthy  non-obese group |  | reference |  |  | reference |  | |  | | reference | |  | |  |
| -Metabolically healthy  obese group |  | 0.70  (0.64-0.78) | <0.001 |  | 0.89  (0.81-0.98) | 0.01 | |  | | 0.88  (0.80-0.97) | | 0.01 | |  |
| -Metabolically unhealthy  non-obese group |  | 1.46  (1.35-1.58) | <0.001 |  | 1.47  (1.34-1.60) | <0.001 | |  | | 1.46  (1.33-1.59) | | <0.001 | |  |
| -Metabolically unhealthy  obese group |  | 0.97  (0.90-1.04) | 0.39 |  | 1.33  (1.23-1.44) | <0.001 | |  | | 1.32  (1.22-1.43) | | <0.001 | |  |

Model 1 adjusted for age and sex. Model 2 further adjusted for comorbidities (coronary artery disease, cerebrovascular disease, left ventricular hypertrophy, and peripheral arterial disease).

Metabolically healthy non-obese group defined as individuals without obesity and metabolic syndrome.

Metabolically healthy obese group defined as individuals with obesity but not concurrent metabolic syndrome.

Metabolically unhealthy non-obese group defined as individuals with metabolic syndrome but not concurrent obesity.

Metabolically unhealthy obese group defined as individuals with both metabolic syndrome and obesity.
