# Supplementary material for: The magnitude of obesity and metabolic syndrome among diabetic chronic kidney disease population: A nationwide study
Source: PLoS One. 2018 May 9;13(5):e0196332. doi: 10.1371/journal.pone.0196332 (PMC5942778; doi:10.1371/journal.pone.0196332)
Supplement: S3 Table — (DOCX) [file pone.0196332.s003.docx]

**S3 Table.** Unadjusted and adjusted odd ratio (OR) and 95% confidence interval (CI) for the association of metabolic syndrome using the Joint Statement criteria and its individual component with the presence of CKD.

| **Methods** |  | **Unadjusted** | |  | **Model 1** | |  | | **Model 2** | | | |  |
| --- | --- | --- | --- | --- | --- | --- | --- | --- | --- | --- | --- | --- | --- |
|  |  | OR  (95% CI) | P |  | OR  (95% CI) | P |  | | OR  (95% CI) | | | P |  |
| **The presence of MetS and its individual component**† | | | | | | | | | | | | | |
| -Metabolic syndrome* |  | 1.30  (1.23-1.38) | <0.001 |  | 1.45  (1.37-1.55) | <0.001 | |  | | 1.44  (1.36-1.54) | <0.001 | |  |
| -WC >90 cm in men and  >80 cm in women |  | 0.97  (0.92-1.03) | 0.29 |  | 0.99  (0.93-1.05) | 0.68 | |  | | 0.97  (0.92-1.04) | 0.52 | |  |
| -blood pressure  ≥ 130/85 mmHg |  | 2.39  (2.19-2.62) | <0.001 |  | 1.57  (1.43-1.73) | <0.001 | |  | | 1.55  (1.40-1.70) | <0.001 | |  |
| -serum triglyceride level  ≥ 150 md/dL |  | 1.41  (1.34-1.48) | <0.001 |  | 1.65  (1.57-1.75) | <0.01 | |  | | 1.65  (1.56-1.74) | <0.001 | |  |
| -serum HDL-C <40 mg/dL  in men and <50 mg/dL in  women |  | 1.36  (1.29-1.42) | <0.001 |  | 1.34  (1.29-1.43) | <0.01 | |  | | 1.35  (1.28-1.42) | <0.001 | |  |

BMI, body mass index; HDL-C, high density lipoprotein cholesterol; WC, waist circumference

Model 1 adjusted for age and sex. Model 2 further adjusted for comorbidities (coronary artery disease, cerebrovascular disease, left ventricular hypertrophy, and peripheral arterial disease).

*metabolic syndrome was defined as having at least three out of five criteria as the followings: elevated waist circumference (>90 and >80 cm in men and women, respectively), triglyceride≥150 mg/dL, HDL<40 in men or <50 mg/dL in women or receiving lipid-lowering drugs, BP≥130/85 mmHg or receiving anti-hypertensive drugs, and blood sugar ≥100 mg/dL or being treated for elevated blood glucose.

†Since all patients were type2 diabetes, we did not analyze the ORs for elevated plasma glucose criterion of metabolic syndrome.
